# 孤立性肺结节恶性概率预测模型的研究进展及临床应用

**DOI:** 10.3779/j.issn.1009-3419.2021.102.29

**Published:** 2021-09-20

**Authors:** 昭珏 王, 静 赵, 孟昭 王

**Affiliations:** 100730 北京，中国医学科学院，北京协和医学院，北京协和医院呼吸与危重症医学科 Department of Respiratory and Critical Care Medicine, Peking Union Medical College Hospital, Chinese Academy of Medical Sciences and Peking Union Medical College, Beijing 100730, China

**Keywords:** 模型, 肺结节, 肺肿瘤, Models, Pulmonary nodules, Lung neoplasms

## Abstract

随着计算机断层扫描（computed tomography, CT）的普及，孤立性肺结节（solitary pulmonary nodule, SPN）发病率明显上升。肺结节良恶性评估对肺结节诊治决策至关重要。许多肺结节恶性概率预测模型已被开发，这些模型基于患者临床和影像学特征来评估肺结节恶性概率。近年来，肺结节恶性概率预测模型在国内逐渐引起关注。本文基于肺结节恶性概率预测模型研究，重点关注在中国患者群体中模型的建立或验证情况，综述肺结节恶性概率预测模型的研究进展和临床应用情况，并为模型未来发展提出思路。

## Abstract (en)

With the popularization of computed tomography (CT) examinations, the incidence of solitary pulmonary nodules (SPNs) has increased significantly. The assessment of benign and malignant pulmonary nodules is crucial to the diagnosis and treatment of lung nodules. Many models for predicting the malignant probability of lung nodules have been developed. These models assess the malignant probability of lung nodules based on the clinical and imaging characteristics of patients. In recent years, malignant probability prediction models have gradually attracted attention in China. Based on the researches on the malignant probability prediction model of pulmonary nodule, focusing on the establishment or verification of the model in the Chinese patient population, this paper reviews the research progress and clinical application of the malignant probability prediction model of pulmonary nodule, and proposes ideas for the future development.

## 1 前言

孤立性肺结节（solitary pulmonary nodule, SPN）是指肺内单发的、被含气肺组织完全包围、界限相对清楚、直径≤3 cm、影像不透明的病变，不伴有肺不张、肺门增大、胸腔积液等表现。近年来，随着电子计算机断层扫描（computed tomography, CT）的日益增多和普及，孤立性肺结节发病率明显上升。肺结节诊治过程中，对结节良恶性的判断至关重要。多种因素已被证实和肺结节恶性有关，包括患者年龄、性别、结节的影像学征象、肿瘤标志物等。而在临床工作中，结节良恶性主要依据医生的经验判断。为此，一些学者基于临床数据，开发了众多肺结节恶性概率预测模型。目前，肺结节恶性概率预测模型多采用多因素*Logistic*回归方法构建，是一种应用于临床医学领域的统计学及经典机器学习的方法。

理想的肺结节恶性概率预测模型，在应用于目标患者群时，应具有较高的预测准确性，通过预测给患者带来获益，指导医生临床工作，并可推广到更广泛群体。模型的预测准确性可通过受试者工作特征曲线（receiver operating characteristic curve, ROC curve）的曲线下面积（area under curve, AUC）衡量，较高的AUC表示该模型具有较高的区分能力。模型预测的病例数量和观察到的病例数量的比值，越接近1.0，准确度越高，低于和高于1.0的值分别表示低估和高估恶性肿瘤风险。决策曲线分析法（decision curve analysis）权衡了准确判断带来的获益和错误判断带来的风险，给出模型在不同风险阈概率的临床获益，近年来也逐渐应用于模型评价。临床医生可对结节恶性概率进行分级评估，计算相应ROC曲线下面积及绘制决策曲线，以此将临床医生判断与模型判断比较。由于群体特征差异，肺结节恶性概率模型在广泛应用时，可能不如在开发模型的人群中准确率高，因此，模型需要进行外部验证，且最好是在大样本、多中心、多样化的肺结节患者群体中验证。

目前，关于肺结节恶性预测模型的研究非常多，且以国外研究居多。本文拟综述国内外常用的肺结节良恶性预测模型，尤其是关注在中国患者群体中建立或验证的肺结节恶性预测模型及临床应用价值，并对未来发展进行展望。

## 2 经典模型及外部验证

### 2.1 国外经典模型

梅奥模型（Mayo model）^[1]^是第一个用于评估结节恶性概率的预测模型，由梅奥医学中心的Swensen等^[1]^在1997年基于多因素*Logistic*回归分析方法建立。Swensen等^[1]^回顾性纳入了1984年1月1日-1986年5月1日胸片发现4 mm-30 mm孤立性肺结节的629例患者，排除了5年内有恶性肿瘤史、既往肺部肿瘤史、恶性表现的患者。纳入分析的因素包括：①病史特征：年龄、性别、吸烟史、戒烟时间、胸外恶性肿瘤史、石棉暴露史、弥漫性间质性肺病史以及阻塞性肺疾病史；②发现结节后的初次肺部CT特征：位置、直径、空洞、支气管充气征、边缘光滑、胸膜牵拉征、不符合良性表现的钙化、卫星病灶、背景中无其他钙化结节、肺部肉芽肿结节、淋巴结增大、淋巴结钙化数量及大小、胸腔积液。经多因素*Logistic*回归分析得6个预测因子，Mayo模型方程为：恶性概率为P=e^x^/（1 + e^x^），x=-6.827, 2 +[0.039, 1×年龄（年）]+（0.791, 7×吸烟史）+（1.338, 8×恶性肿瘤史）+[0.127, 4×直径（mm）]+（1.040, 7×毛刺）+（0.783, 8×上叶），在学习集及验证集的AUC分别为0.833和0.801。Mayo模型的学习集和验证集均来自于同一个良性比例较高的患者群体（65%为良性，23%为恶性，12%无法确定），且以发现结节时初次CT为影像学基础。综上两点，Mayo模型适用于较为广泛的偶发肺结节患者在诊断时的恶性概率风险评估。该模型主要缺点是对于部分结节良恶性的诊断不够明确，导致模型准确性欠可靠。由于该研究为前瞻性研究，具有有限的随访时间，而肺部恶性结节，尤其是肺腺癌早期或其癌前病变，可能具有长达近十年的惰性时期，因此部分恶性结节诊断不明，甚至可能被诊断为良性结节，此缺点为肺结节模型前瞻性研究的固有缺陷。Herder等^[2]^在2005年对Mayo模型进行改进，采用相同研究方法，加入了正电子发射计算机断层显像（positron emission tomography computed tomography, PET-CT）中结节摄取作为一个预测因素，建立了Herder模型。Herder模型在肿瘤患病率为57%肺结节患者队列中AUC值为0.88，明显优于Mayo模型。

Brock模型（Brock model），也被称作PanCan模型、McWilliams模型^[3]^，是由McWilliams等^[3]^在2013年使用泛加拿大早期肺癌检测研究（Pan-Canadian Early Detection of Lung Cancer Study）患者数据建立，亦采用多因素*Logistic*回归分析方法。研究者排除了无吸烟史、既往肿瘤史、年龄 < 50岁或年龄>75岁患者，共有1, 871例（7, 008个结节）纳入，恶性率为1.4%，结节直径为（4.3±3.7）mm。所有患者有病理结果为诊断标准，整理其病史和基线低剂量CT，建立Brock模型方程：x=- 6.614, 4 +（0.646, 7×性别）-[5.553, 7×直径（mm）]+（0.930, 9×毛刺）+（0.600, 9×上叶）。研究者在英属哥伦比亚癌症机构（British Columbia Cancer Agency）进行了外部验证，验证集亦是一个低恶性率（42/5, 021=0.8%）、平均直径小于（3.7±2.5）mm的群体。Brock模型在其学习集及验证集的AUC均非常高，分别为0.942和0.970。Brock模型特点有：①所有患者均有吸烟史，年龄在50岁-75岁之间，无既往肿瘤史，此类群体为肺癌高危人群，为在常规体检中通过CT进行肺癌筛查的典型群体；②总恶性概率低（< 2%），肺结节平均直径小（< 0.5 cm），亦接近体检发现肺结节的患者群体特征；③Brock模型是目前唯一基于基线低剂量CT开发的模型，低剂量CT是体检肺癌筛查使用的常规检查。综上，Brock模型非常适用于体检发现肺部结节患者在诊断时的恶性概率评估。Brock模型亦具有较高的可靠性。从研究过程而言，所有患者均为病理确诊，诊断明确，无由于诊断错误或诊断不明带来的偏差。从外部验证而言，Brock模型的外部验证在来自于另一国家完全独立的验证集进行，Brock模型在验证集中的优秀表现表明该模型受到区域的影响很小，具有在广泛地区准确预测的潜力。

较为经典的国外模型还有退伍军人模型（VA model）^[4]^，由Gould等^[4]^在2007年使用美国退伍军人事务部（Department of Veterans Affairs, VA）的数据以相同统计学方法建立。VA模型以X线作为影像学检查，98%为男性，恶性患病率为54%。模型预测因子包括吸烟史、年龄、结节直径、戒烟时间，AUC值为0.78。VA模型以X线而非CT作为影像学基础，不能反映结节形态细节，因此准确性较低。此外，VA模型在女性中应用受限。由于胸部CT已广泛应用于肺结节的诊断和评估，肺结节在女性患者中发病亦普遍，VA模型在目前临床中意义不大。

### 2.2 国内经典模型

最早开发的模型为PKUPH模型^[5]^，也是目前最受接受及广泛验证的模型。李运等^[5]^纳入了北京大学人民医院371例经手术切除的孤立性肺结节患者作为学习集，恶性率为53.1%，建立多因素*Logistic*回归数学模型：P=e^x^/（1+e^x^），x=-4.496+（0.070×年龄）+（0.676×肿瘤最大径) +（0.736×毛刺征）+（1.267×肿瘤家族史）-（1.615×钙化）-（1.408×边界清楚）。该模型在一纳入67例患者的独立验证集中AUC值为0.888±0.054。该研究为首个在国内进行的，同时考虑了病史及影像学资料，数据较完备的肺结节模型研究，适用于中国患者。然而，研究者未报道建模过程使用的是术前末次CT还是诊断时首个CT，难以确定模型适用时机。

国内另一早期建立的经典模型为PUMC模型^[6]^，由Dong等在2013年建立，亦采用相同方法。该模型的学习集为中国医学科学院肿瘤医院1, 679例手术切除的孤立性肺结节患者，恶性率为77.2%，该模型包含多项预测因素：年龄、癌胚抗原（carcinoembryonic antigen, CEA）、细胞角蛋白19片段抗原（cytokeratin 19 fragment antigen 21-1, CYFRA21-1）、吸烟史、肿瘤家族史、结节直径、结节边界清晰、卫星灶、分叶征、钙化、毛刺征，模型在学习集和来自同一中心的验证集中的AUC值为0.935和0.917。PUMC模型主要特点在于：①除病史资料、胸部CT表现外，还纳入了5项肺癌相关肿瘤标志物[CEA、神经元特异性烯醇化酶（neuron-specific enolase, NSE）、CYFRA21-1、糖类抗原125（carbohydrate antigen 125, CA125）、鳞状细胞癌抗原（squamous cell carcinoma antigen, SCC-Ag）]作为分析因素；②患者在术前30 d内进a行胸部CT和血清学检查；③患者恶性率远远高于其他模型。因此，与Brock模型主要用于在筛查群体中区分恶性结节相反，PUMC模型适用于恶性可能性较高、已经完善肿瘤标志物检查、面临手术决策的患者，将良性结节患者从其中区别出来。

### 2.3 经典模型的外部验证

以上模型在国内患者群体中均进行过外部验证^[7-19]^，其中Mayo模型、VA模型、Brock模型、PKUPH模型是国内肺结节恶性概率预测模型验证研究中最感兴趣的模型。表 1总结了一些外部验证研究。这些外部验证均在恶性概率较高（48.8%-86.5%）的患者群体中进行^[7-19]^，多数研究中患者有基于手术切除或活检、细针穿刺活检或细胞学病理诊断的结果。目前，国内北方地区的研究少于南方地区，最多的研究集中在沿海区域。

**表 1:** Mayo模型、VA模型、Brock模型和PKUPH模型在国内的外部验证研究 External verification of Mayo model, VA model, Brock model and PKUPH model in China

Central location	Malignant rate	AUC			
		Mayo model	VA model	Brock model	PKUPH model
Sichuan^[7]^	76.0%	0.705	0.646	0.575	0.675
Hubei^[8]^	78.4%	0.626	0.621	0.600	0.630
Guangdong^[9]^	60.0%	0.752	0.730	0.878	0.833
Beijing^[10]^	86.5%	0.739	0.715	0.709	0.755
Jiangsu^[11]^	86.4%	0.597	0.538	0.430	0.623
Xi’an^[12]^	48.8%	0.655	0.603		0.521
Zhejiang^[13]^	67.2%	0.789	0.746		
Chongqing^[14]^	68.0%	0.649	0.599		
Guangdong^[15]^	81.2%	0.753	0.728		0.800
Shanghai^[16]^	78.7%	0.701	0.729		0.773
Jiangsu^[17]^	55.0%	0.764	0.715		
Guangdong^[18]^	68.9%	0.685			0.646
Jiangxi^[19]^	56.7%	0.745			0.825
AUC: area under the curve					

在这些研究中，各个模型对于肺结节良恶性的诊断效能均低于在开发队列中的效能。Mayo模型、VA模型、Brock模型和PKUPH模型的AUC面积分别为0.597-0.789^[7-19]^、0.600-0.728^[7-17]^、0.430-0.878^[7-11]^和0.521- 0.833^[7-12, 15, 16, 18, 19]^，均低于它们在开发队列中的AUC值（0.88, 0.78, 0.94, 0.89）^[1, 3-5]^。

各模型间比较，PKUPH模型和Mayo模型诊断效能较好。Brock模型仅在一项研究中AUC高于其余所有模型^[9]^，其表现不佳可能是由于Brock模型更适用于恶性肿瘤患病率低的群体。VA模型准确性低，主要原因在于以X线作为影像学检查、基于男性为主开发。令人惊讶的是，尽管PKUPH模型建立在恶性率相近的国内患者数据基础上，Mayo模型建立时的患者群体和国内患者群体差异较大，PKUPH模型和Mayo模型在国内患者的外部验证中，结果并无明显差异^[7-12, 15, 16, 18, 19]^。PUMC模型外部验证极少。原因可能在于PUMC模型纳入了肺癌相关肿瘤标志物CEA、CYFRA 21-1作为预测因素，普适性受限。

## 3 国内开发的其他模型

国内研究者基于国内患者队列开发肺结节恶性概率预测模型始于2010年左右。自2010年来，不同中心均在自己的患者队列中开发了几十个模型^[11, 16, 17, 20-30]^，覆盖我国北部、西北、东南、西南等地域。除了肺部CT外，肿瘤标志物和PET-CT等检查更多地被纳入研究。大多数研究仍为回顾性、单中心、应用*Logistic*回归方法建立模型，分析患者临床资料、影像学表现（胸部CT为主），部分研究纳入PET-CT和血清学检查。在这些模型中，除个别研究有一个外部验证外，均无外部验证研究支持。

国内有一个跨地区的多中心的肺结节恶性概率模型研究^[31]^。由Yang等^[31]^在2018年开发，该研究纳入来自北京、河南、南京、上海、重庆5个中心的共715例孤立性肺结节患者。Yang等^[31]^亦使用*Logistic*回归方法，使用393例患者作为训练集，建立了包含患者的临床数据、肺部CT表现、肿瘤标志物的模型，其预测因素为：年龄、吸烟史、结节直径、毛刺征、性别、胃泌素释放肽前体（Progastrin-releasing peptide, ProGRP）、SCC-Ag、CYFRA21-1、CEA。由于此项研究跨越国内多个地区，该模型具有应用于全国各地区患者的潜力。然而，虽然模型在训练集中表现出良好的诊断效能（AUC=0.915, 1），在验证集中，模型的诊断能力十分有限（AUC=0.583, 6）。因此，该模型实际能否在全国应用，仍需要更多外部验证结果。另一方面，可惜的是，在这个国内多中心的患者队列中，并未进行其他模型的验证。

众多以*Logistic*回归方法建立的预测模型所纳入的独立预测因素有许多不同。2019年，张凯等^[32]^对于基于国内人群的肺癌恶性概率预测模型进行了一项*meta*分析，共回顾了2010年-2018年的18项研究，结果显示，人口学特征4个变量（年龄、家族史、既往肿瘤史、吸烟史）、影像学特征8个变量（毛刺症、结节直径、分叶、毛玻璃样、边界模糊、胸膜凹陷征、短毛刺、最大标准摄取值（Maximum standardized uptake value, SUVmax）、血清学1个变量（CYFRA21‐1）为导致SPN恶性的危险因素，影像学特征2个变量（钙化、边界清楚）为SPN恶性的保护因素。但该*meta*的文献检索策略欠完善，可能存在漏检。尽管如此，该研究结果中出现了多个人口学变量和影像学变量，这些变量出现在部分模型中，但在另一些模型中未出现。这说明这些变量对于肺部结节恶性程度预测事实上的贡献无巨大差异，而不同肺结节恶性预测模型的不同结果主要来自于样本来源相关偏倚。因此，以更少的变量构建准确的、普适的肺结节恶性概率预测模型可能存在较大困难。

## 4 各种预测模型在临床中的实用情况

将预测模型和临床医生判断的准确程度加以比较是衡量模型实用性的另一角度。国内有一项研究^[33]^比较了放射科医生和Mayo模型、Brock模型、退伍军人模型诊断肺结节效能。研究纳入了277例患者，恶性率为74.7%，以病理学结果或随访两年有无变化作为良恶性诊断依据。放射科医生将结节风险评估为5个等级：良性、可能良性、不确定、可能恶性、高度怀疑恶性。放射科医生、Mayo模型、Brock模型的ROC曲线下面积无显著差异，但退伍军人模型显著低于三者。决策曲线分析显示放射科医生评估比三个模型均带来更高的获益。国外此类研究稍多^[34-36]^，但预测模型的表现均不优于临床医生。

目前，国内外不同肺结节诊治指南均在其临床路径中提到恶性概率模型^[37-42]^。指南和共识提供肺结节诊治的临床路径，首先应用少量危险因素（如年龄、吸烟史、结节直径、是否是实性结节）将患者进行风险分层，并对每一分层提出相应的、具有可操作性的临床管理路径（如：3个月后随访、6个月后随访、年度随访、活检明确诊断、手术治疗等）。与之不同，恶性概率模型选择更多的危险因素、以定量方式评估该结节的恶性概率，进一步指导临床医生对每个个案做出临床决策。

通过肺结节诊治路径的危险分层后，仍有部分患者的临床决策在恶性风险和有创操作风险的衡量之间难以取舍。对于此类患者，指南或共识推荐临床医生可使用肺结节恶性概率预测模型为临床决策提供参考。结合目前预测模型的外部验证情况，指南还强调模型选择问题和临床医生判断的价值。

美国胸科医师协会（American College of Chest Physicians, ACCP）2013年肺结节诊治指南^[37]^，提到包括梅奥模型、VA模型等多个模型，但表明模型整体准确率并不高于专科医生，建议依据目标人群特点、易用性及外部验证程度选择模型，但未推荐具体选择哪个模型。同时，由于模型与临床医生判断间相关性较差，指南指出模型可能能为临床医生提供独特的信息。

美国国家综合癌症网络（National Comprehensive Cancer Network, NCCN）2021年更新的肺结节筛查指南中^[38]^，恶性概率预测模型仅仅作为多学科团队对于中高风险结节评估的一部分，最终由多学科团队共同决定患者是继续进行3个月或6个月的影像学随访还是进行活检或手术治疗。这些患者包括：部分首发≥8 mm的实性结节、部分首发且实性成分≥6 mm的部分实性结节、随访无增大的≥15 mm的实性结节、随访有变化的≥8 mm的部分实性结节。指南同时强调多学科团队的重要性，强调模型不能取代多学科团队的作用。

亚洲肺结节诊治共识^[39]^中，临床医生对结节恶性风险的判断会影响直径>8 mm实性结节的处理方式。共识指出，欧美模型并不一定能适用于亚太地区。专家组建议，无论是否使用模型，临床医生应当决定后续策略。

我国肺结节诊治专家共识（2018版）^[40]^也强调模型的适用性问题。比如，亚太地区为结核高发地区，而结核亦好发于上叶，因此肺结节位于上叶作为一个预测因素并不适合亚太地区使用。该共识亦未推荐具体模型，并建议依据目标人群特点、易用性及验证程度选择模型。然而，根据本文总结，实际上在国内经过一般性检查（即病史采集和CT检查）的患者中，并无经过验证仍较理想的模型可供选择。肺癌筛查与管理中国专家共识^[41]^中提到Yang等在2018年经多中心研究建立的模型，适用于中国高风险人群。然而，该模型本身尚未经过更多的外部验证。

Fleischner学会作为放射科学会^[42]^，其肺结节指南中并未提到临床恶性概率预测模型。

## 5 基于人工智能的肺结节恶性概率预测

应用人工智能进行肺结节恶性预测研究近几十年来均在持续进行。人工智能主要基于两种策略完成结节分类，一是经典机器学习技术，二是深度学习。深度学习整合了肺结节特征提取和分类两个步骤，直接从CT影像得出良恶性分类结论，为头到头的黑箱学习形式。目前，深度学习表现优于机器学习，不少研究显示出优良的准确率^[43, 44]^。卷积神经网络（convolutional neural network, CNN）是深度学习中用来预测恶性肺结节的主要类型。

关于人工智能在肺结节良恶性判断上的应用，有以下几方面不足。

首先，机器学习的目标是良恶性二分类，除了采用*Logistic*回归方法等特定算法以外，均只给出良恶性分类结果，不能给出恶性概率。*Logistic*回归方法作为统计学及经典机器学习方法，可在经典机器学习策略中应用，但此策略在建模方法上无本质变化，难以带来模型准确性的突破性提高。目前表现更加突出的深度学习方法只能得出分类结果。

其次，迄今为止，无论是基于经典机器学习策略或是深度学习，绝大多数研究仅仅提取了影像学特征作为预测因子，而未考虑病史和血清学资料。国内有一此类研究^[45]^，但亦仅仅给出分类结果。该研究使用了包括*Logistic*回归在内的5种传统深度学习方法[*Logistic*回归（*Logistic* regression, LR）、人工神经网络（artificial neural network, ANN）、k-邻近算法（k-nearest neighbor, KNN）、支持向量机（support vector machines, SVM）、随机森林（random forest, RF）]，以388例患者的病史信息、CT表现和血清肿瘤标志物为基本资料，构建了5种恶性概率预测模型。这5种模型在验证集中的曲线下面积均高于Mayo模型，其中SVM模型和LR模型表现较好。

最后，机器学习提取的影像学特征不同于传统临床影像学征象。传统影像学征象可从病理学角度理解，因此能够在实际和其他良性结节鉴别时有鉴别诊断意义，所以可通过较小样本量的学习获得较可靠诊断模型。人工智能图像识别的影像学特征尚无明确病理意义，当相应模型应用于未能被训练集大量覆盖的良性结节病例时，误判可能较大。

基于人工智能进行肺结节恶性概率预测，可从以下几方面设想：①影像学特征选择：寻找适合于人工智能的影像学特征，可以训练人工智能识别传统影像学征象，或从目前人工智能常用的影像学特征中筛选可理解的特征；②将影像学特征与其他临床信息共同纳入模型开发；③应用其他概率模型。

## 6 未来研究方向

肺结节恶性概率预测模型，旨在帮助临床对于肺结节患者权衡随访或有创检查的获益，指导肺结节诊治。综上，无论是初诊肺结节患者还是肺结节术前患者，目前国内尚无经过可靠外部验证、可应用于国内各地区、预测准确性良好的模型。仍有大量研究值得进一步深入，包括：①尚无恶性程度更低的肺结节患者中模型的建立和验证研究，如体检人群，在此类群体中，Brock模型的验证仍值得期待；②尚无理想的覆盖国内多地域的多中心研究，包括模型的建立和验证；③模型和临床医生预测能力的探索仍需进行，尤其是与基层医疗中心执业医师、全科医师等对比研究十分重要，这将明确此类模型在指导广泛基层医疗、而非肺结节中心医疗的意义；④现有基于*Logistic*统计回归方法开发的模型，前景并不乐观，基于人工智能、应用其他方法的模型值得尝试。
